# Genetic deletion of soluble epoxide hydrolase delays the progression of Alzheimer’s disease

**DOI:** 10.1186/s12974-019-1635-9

**Published:** 2019-12-17

**Authors:** Hsueh-Te Lee, Kuan-I Lee, Chia-Hui Chen, Tzong-Shyuan Lee

**Affiliations:** 1 Institute of Anatomy and Cell Biology, National Yang-Ming University, Taipei, Taiwan; 2 Department of Physiology, National Yang-Ming University, Taipei, Taiwan; 3 Graduate Institute and Department of Physiology, College of Medicine, National Taiwan University, Taipei, 10051 Taiwan

**Keywords:** Soluble epoxide hydrolase, Alzheimer’s disease, Inflammation, Astrocyte

## Abstract

**Background:**

Soluble epoxide hydrolase (sEH) is a bifunctional enzyme with COOH-terminal hydrolase and NH2-terminal lipid phosphatase activities. It is expressed in various cell types in the brain and is involved in the pathogenesis of inflammatory and neurodegenerative diseases. Alzheimer’s disease (AD) is a progressive neuroinflammatory and neurodegenerative disease. However, the pathological significance of sEH and underlying molecular mechanism in AD remain unclear.

**Methods:**

To examine the role of sEH in pathogenesis of AD, we used wild-type (WT) mice, soluble epoxide hydrolase deficient (*sEH*^−/−^) and two mouse models of AD, including amyloid precursor protein (APP)/presenilin 1 (PS1) transgenic (*APP/PS1* Tg) and *APP/PS1* Tg/*sEH*^−/−^ mice. Western blotting analysis and immunohistochemistry assay were performed to evaluate the protein expression. Locomotion, nesting building ability, Y-maze, and Morris water maze tests were conducted to study mouse behavior. The levels of interleukin (IL)-1β, IL-4, IL-6, and IL-10 and the activities of NF-κB and nuclear factor of activated T cells (NFAT) were measured by commercial assay kits. The quantitative protein level profiling in the brain lysate was analyzed using LC-MS/MS approaches.

**Results:**

We demonstrated that the level of sEH was increased in the brain and predominantly appeared in hippocampal astrocytes of *APP/PS1* Tg mice. Genetic ablation of *sEH* in *APP/PS1* Tg mice delayed the progression of AD as evidenced by the alleviation in behavior outcomes and Aβ plaque deposition. In addition, loss of the function of *sEH* in *APP/PS1* Tg mice increased astrogliosis and the production of astrocyte-derived anti-inflammatory cytokines including IL-1β, IL-4, and IL-10, as well as the activity of NF-kB and NFAT. Moreover, analysis of gene ontology in the AD brain revealed that important signaling pathways and processes related to AD pathogenesis such as translational regulation, oxidative stress, cytoskeleton reorganization, and small GTPase signal transduction were altered in *APP/PS1* Tg/*sEH*^−/−^ mice compared with *APP/PS1* Tg mice.

**Conclusion:**

Our results suggest that sEH is a crucial regulator in the progression of AD and might be a potential therapeutic target for the treatment of AD.

## Introduction

Soluble epoxide hydrolase (sEH), an enzyme with COOH-terminal hydrolase (EH) and NH2-terminal lipid phosphatase (PT) activities, is expressed in several tissues, including the brain, heart, and kidney [1–4]. In the brain, sEH is predominantly expressed in cortex, hippocampus, white matter, substantia nigra, and striatum [4–6]. Apart from the endothelial cells (ECs) of vessels, sEH appears in neurons and glial cells [4, 7]. The precise role of sEH in the pathogenesis of inflammatory and brain diseases has been well defined [8, 9]. Recent studies demonstrated that inhibition of the EH activity of sEH confers protection from ischemia-induced brain injury in rodents, suggesting that sEH is a crucial mediator in cerebrovascular and neuronal function upon the pathological insults [6, 7, 10–13]. In addition, several studies indicated the potential function of sEH in age-related cognitive decline and neurodegenerative disease [14–16]. However, the role of sEH and its underlying mechanism in neurodegenerative diseases are not fully understood.

Alzheimer’s disease (AD) is a progressive neurodegeneration disease with typical symptoms, including impairment of learning, memory, and cognition abilities [17, 18]. Recently evidence has indicated that amyloid-β (Aβ) plaque deposition is one of the pathological hallmarks in AD [17, 19, 20]. The Aβ peptide is generated from amyloid precursor protein (APP) by β-site APP cleavage enzyme 1 (BACE1) and presenilin 1 (PS1) [19, 21]. Particularly, neurotoxic Aβ triggers inflammation in glial cells and induces neurodegeneration, ultimately leading to the progression of AD [19, 21–24]. Moreover, astrocyte-derived inflammatory responses play a crucial role in AD pathology [25, 26]. Upon Aβ stimulation, reactive astrocytes can release pro-inflammatory cytokines including interleukin (IL)-1β, IL-6, and anti-inflammatory cytokines, IL-4 and IL-10 [24, 27]. Two different transcription factors NF-kB and nuclear factor of activated T cells (NFAT) are involved in the astrocyte-mediated cytokines production; NF-κB regulates the production of IL-1β and IL-6, and NFAT regulates the expression of IL-4 and IL-10 production [28–31]. However, the critical role and the molecular mechanisms underlying astrocyte-mediated inflammatory response upon Aβ stimulation are not fully elucidated.

In this study, we aimed to investigate the potential role of sEH and possible molecular mechanisms during AD pathogenesis by using the mouse models. Here, we report that the protein level of sEH was increased in astrocytes of 6-month-old *APP/PS1* Tg mice. We demonstrated that genetic deletion of *sEH* in the *APP/PS1* Tg mice rescued the impairment of AD pathologies, including Aβ plaque deposition, cytokines production, and dysfunction of behavioral outcomes. The quantitative proteomic analysis of brain samples from *APP/PS1* Tg/*sEH*^−∕−^ and *APP/PS1* Tg mice was performed by liquid chromatography-tandem mass spectrometry (LC-MS/MS). Mapping of the deregulated proteins with bioinformatics tools revealed that differentially abundant proteins were significantly enriched for pathways and processes known to be important in AD pathology, including translational regulation, oxidative stress, cytoskeleton reorganization, and small GTPase-mediated signal transduction. Collectively, our results suggest that sEH is a key regulator of astrocytes-derived inflammation in AD progression.

## Methods

### Reagents

Rabbit anti-sEH (sc-25797), mouse anti-von Willebrand factor (vWF) (sc-365712), goat anti-rabbit IgG FITC-conjugated (sc-2012), Texas red-conjugated (sc-2780), and goat anti-mouse IgG Texas red-conjugated (sc-2781) antibodies were obtained from Santa Cruz Biotechnology (Santa Cruz, CA, USA). Rabbit anti-glial fibrillary acidic protein (GFAP) (AB5804), mouse anti-GFAP (MAB360), anti-NeuN (MAB377B), and anti-ionized calcium-binding adapter molecule 1 (IBA-1) (MABN92) antibodies were obtained from Millipore (Darmstadt, Germany). Rabbit anti-LDLR-related protein 1 (LRP-1) (L2170) antibody, mouse anti-α-tubulin (T9026) antibody, bovine serum albumin (BSA), and phosphatase inhibitor cocktails 1 and 2 were obtained from Sigma-Aldrich (St. Louis, MO, USA). Mouse anti-Aβ (SIG-39320) antibody was from Covance (Dedham, MA, USA). Rabbit anti-β-APP C-terminal fragment (βCTF) (802801) antibody was obtained from BioLegend (San Diego, CA, USA). Mouse anti-ATP-binding cassette transporter A1 (ABCA1) (ab18180), rabbit anti-BACE1 (ab2077), anti-IL-1β (ab9722), anti-IL-4 (ab9622), anti-IL-6 (ab6672), and anti-IL-10 (ab9969) antibodies were obtained from Abcam (Cambridge, MA, UK). Rabbit anti-apolipoprotein E (apoE) (1930-1) antibody was obtained from Epitomics (Burlingame, CA, USA). Retrieval buffer was from Biocare Medical (Concord, CA, USA). The mounting medium with DAPI was from Vector Laboratories (Burlingame, CA, USA). The ELISA kit for NF-κB activity was from Cayman Chemical (Ann Arbor, MI, USA) and for NFAT activity from Active Motif (Carlsbad, CA, USA). ELISA kits for IL-1β, IL-4, IL-6, and IL-10 were obtained from R&D systems (Minneapolis, MN, USA).

### Mice

The investigation conformed to the Guide for the Care and Use of Laboratory Animals (Institute of Laboratory Animal Resources, eighth edition, 2011), and all animal experiments were performed in accordance with the approved guidelines by the Animal Care and Utilization Committee of the National Yang-Ming University. C57BL/6 J WT, Ephx2^tm1/Gon2/J^ (*sEH*^−∕−^) mice, and B6.Cg-Tg(APPswe, PSEN1dE9)85Dbo/J (*APP/PS1* Tg) mice were purchased from Jackson Laboratory (Bar Harbor, ME, USA). For the generation of *APP/PS1* Tg/*sEH*^−∕−^ mice, *sEH*^−∕−^ mice were crossed with *APP/PS1* Tg background, and the genotypes were confirmed by PCR of genomic DNA isolated from these animals. Mice were housed in barrier facilities, maintained in a 12-h/12-h light and dark cycle. Temperature (22 °C) and humidity (40–60%) of the vivarium were tightly controlled. Mice were group-housed 3–4 per cage and fed a regular chow diet, which contained 4.5% fat by weight (0.02% cholesterol) (Newco Distributors, Redwood, CA, USA). At the end of the experiments, mice were euthanized with CO_2_, and then brain tissues were collected for histological analysis or were stored at − 80 °C. The isolated brain tissues were homogenized, and lysates were subjected to western blot analysis.

### Western blot analysis

Brain tissues were lysed in immunoprecipitation lysis buffer (50 mmol/L Tris pH 7.5, 5 mmol/L EDTA, 300 mmol/L NaCl, 1% Triton X-100, 1 mmol/L phenylmethylsulfonyl fluoride, 10 μg/mL leupeptin, and 10 μg/mL aprotinin). Aliquots of brain lysates were separated on SDS-PAGE, transferred to membranes, incubated with primary antibodies, and then horseradish peroxidase-conjugated secondary antibody. Bands were detected using an enzyme-linked chemiluminescence detection kit (Perkin Elmer, Waltham, MA) and the band signal was quantified by Imagequant 5.2 (Healthcare Bio-Sciences, Philadelphia, PA).

### Immunohistochemistry staining

The brain tissues were fixed in 4% paraformaldehyde and 15-μm cross sections were prepared. Sections were incubated with retrieval buffer for 10 min, blocked with 2% BSA for 60 min, and incubated with primary antibody overnight at 4 °C, then FITC- or Texas Red-conjugated secondary antibody for 1 h at 37 °C. Antigenic sites were visualized under a Nikon TE2000-U microscope (Tokyo) with QCapture Pro 6.0 software (QImaging, BC, Canada).

### Measurement of inflammatory cytokines

The concentrations of pro-inflammatory cytokines, including IL-1β, IL-4, IL-6, and IL-10 in brain lysates, were measured by using the ELISA kits according to the manufacturer’s instructions.

### Measurement of DNA-binding activity on NF-κB and NFAT

The DNA-binding activities of NF-κB and NFAT in brain lysates were measured by using the ELISA kit according to the manufacturer’s instructions.

### Nest building

The protocol of nest building has been described [32]. Each mouse was housed in a single cage containing two pieces of cotton (5 × 5 cm) from the first day. The presence and quality of nesting were recorded by the nesting score which separated 5-point scale ranging from 1 to 5 as follows: 1 = nest not noticeably touched, 2 = cotton partially torn up, 3 = mostly shredded cotton, but often no identifiable nest location, 4 = a markedly nesting site, but flat nest, and 5 = a (near) perfect nest. Nesting score was recorded manually at 24 h.

### Y-maze

The protocol of Y-maze was followed as previously described with modifications [33]. The Y-maze apparatus consisted of three arms made of stainless steel joined in the middle to form a “Y” shape. The mice were placed into one of the arms of the maze (star arm) and allowed to explore the maze with only one of the arms closed for 10 min (training trial). After 3 h, mice were returned to the Y-maze by placing them in the start arm. Then, the mice were allowed to explore freely all three arms of the maze for 5 min (test trial). The number of entries into and the distance in each arm, and the first choice of entry were registered from video recordings.

### Morris water maze

The protocol of Morris water maze (MWM) has been described [33] and modified for this study. A large circular tank (diameter 0.8 m and depth 0.4 m) was filled with water (25 ± 1 °C, depth 20 cm) and the escape platform (8 cm × 4 cm) was submerged 1 cm below the surface. The training section was monitored by a video recording system. The escape latency and trajectory of swimming were recorded for each mouse. The hidden platform was located at the center of one of the four quadrants to the pool. The location of the platform was fixed throughout testing. Mice had to navigate using extra-maze cues that were placed on the walls of the maze. From day 1 to day 4, mice went through three trials with an inter-trial interval of 5 min. The mice were placed into the pool facing the sidewall randomly at one of four start locations and allowed to swim until they found the platform, or for a maximum of 2 min. Any mouse that failed to find the platform within 2 min was then guided to the platform. The animal then remained on the platform for 20 s, and then they were removed from the pool. The day after the completion of the hidden platform training, a probe trial was conducted to determine whether mice used a spatial strategy to find the platform. In day 5, the platform was removed from the pool, and the mice were allowed to swim freely for 2 min. The percentage of time spent in each quadrant of the pool and the number of times the mice crossed the former position of the hidden platform were recorded.

### LC-MS/MS analysis

The LC-MS/MS analysis of brain tissues from *APP/PS1* Tg mice and *APP/PS1* Tg/*sEH*^−∕−^ mice was performed by researchers of Visual Protein Company (Taipei, Taiwan).

### Gene ontological analysis

The gene ontological analysis of brain tissues from *APP/PS1* Tg and *APP/PS1* Tg/*sEH*^−∕−^ mice was performed by researchers of Visual Protein Company (Taipei, Taiwan).

### Statistical analysis

Results are presented as mean ± SEM. Mann-Whitney *U* test was used to compare two independent groups. Kruskal-Wallis followed by Bonferroni post hoc analyses were used to account for multiple testing. SPSS v20.0 (SPSS Inc., Chicago, IL) was used for analysis. Differences were considered statistically significant at *P* < 0.05.

## Results

### The level of sEH is upregulated in hippocampal astrocytes during AD progression

**Fig. 1:**
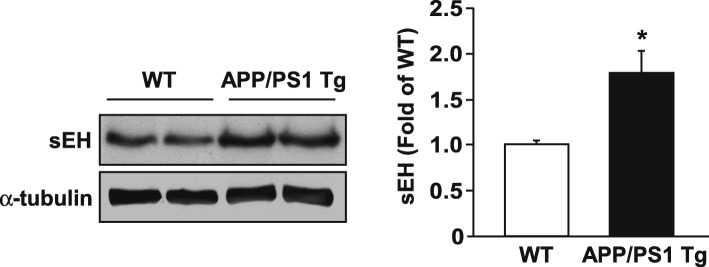
The protein expression of sEH is increased in the brain of *APP/PS1* Tg mice. Brains were harvested from 6-month-old WT mice and *APP/PS1* Tg mice. Western blot analysis of sEH and α-tubulin. Data are mean ± SEM from 6 mice in each group. *P < 0.05 vs. WT mice

**Fig. 2:**
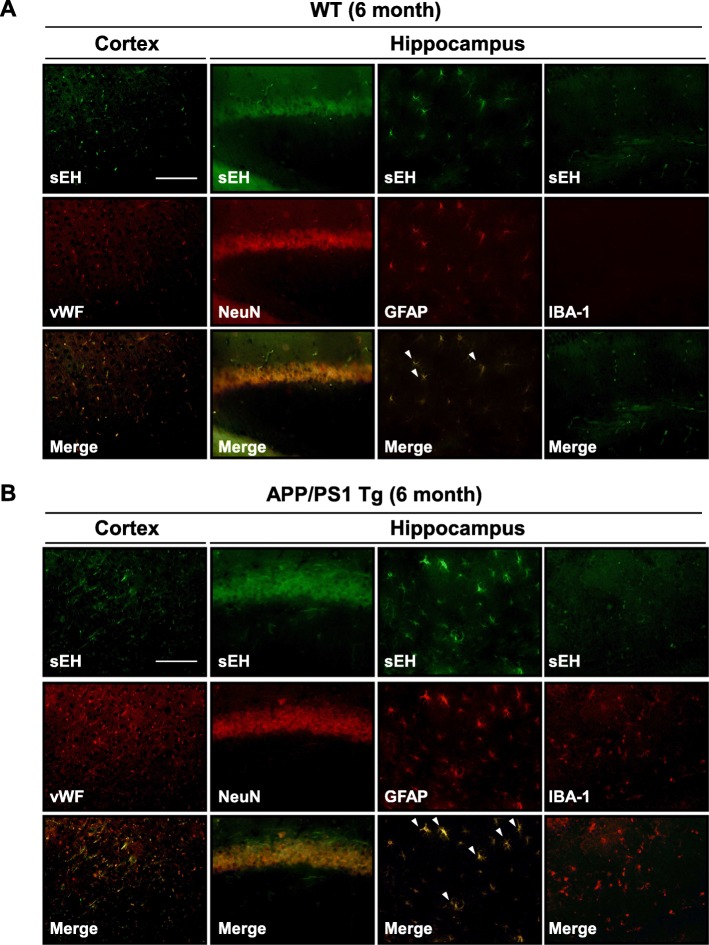
The level of sEH protein is increased in hippocampal astrocytes of *APP/PS1* Tg mice. **a, b** Brain tissue sections from 6-month-old WT mice and *APP/PS1* Tg mice were immunostained with anti-sEH, anti-vWF (for endothelial cells), anti-NeuN (for neurons), anti-GFAP (for astrocytes), anti-IBA-1 (for microglia), and their corresponding secondary antibodies. Scale bar = 50 μm. Arrowheads indicate sEH-positive astrocytes

To explore the role of sEH in the pathogenesis of AD, we first investigated the levels of sEH in mouse brain from WT and *APP/PS1* Tg mice. The results of western blot analysis showed that the protein level of sEH was increased in 6-month-old *APP/PS1* Tg mice (Fig. 1). Immunohistochemistry analysis revealed that sEH was primarily localized in ECs of vessels, hippocampal neurons, and astrocytes in WT or *APP/PS1* Tg mice. However, such an increase in sEH expression was remarkably restricted to astrocytes in the brain of *APP/PS1* Tg mice (*APP/PS1* Tg mice vs. WT mice = 4.23 ± 1.35 vs. 1.14 ± 0.28-fold) (Fig. 2a and b). These data suggest that sEH might play an important role in the development of AD.

### Genetic deletion of *sEH* improves the nesting building ability, learning, and memory in the *APP/PS1* Tg mice

**Fig. 3:**
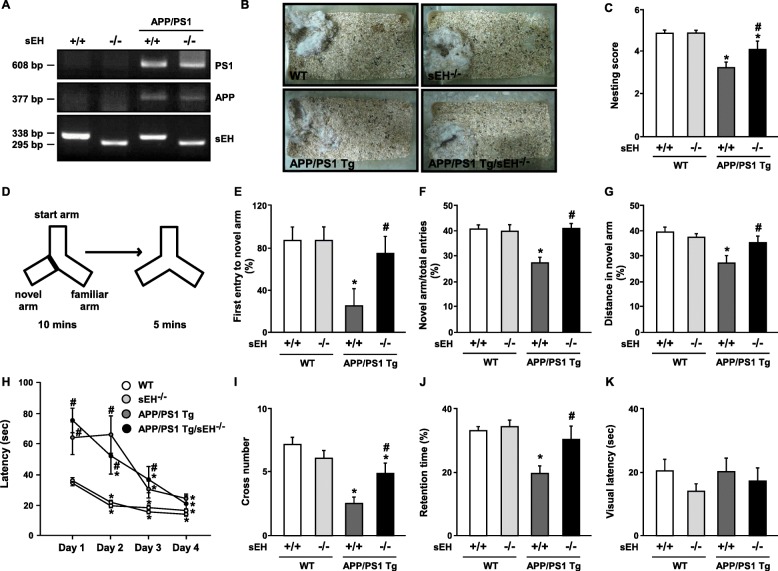
Deletion of sEH recovers nesting building ability, spatial learning, and memory in *APP/PS1* Tg mice. **a** PCR analysis for genotyping of representative animals depicting WT, *sEH*^−∕−^, *APP/PS1* Tg, and *APP/PS1* Tg/*sEH*^−∕−^. PCR bands of 608 bp for PS1, 377 bp for APP, 338 bp for sEH^+/+^, and 295 bp for sEH^−∕−^ were amplified for genotypes. **b**, **c** Representative examples and the score for nest building for 6-month-old WT, *sEH*^−∕−^, *APP/PS1* Tg, and *APP/PS1* Tg/sEH^−∕−^ mice. **d**–**g** Schematic diagram of Y-maze experimental design and ratio of first entry, number of entries, and distance moved in the novel arm. **h** Morris water maze (MWM) test of learning patterns verified on days 1 to 4. **i–j** At day 5 after training, the number of times of crossing the hind platform and retention times in the target quadrants. **k** The latency of arrival to the visible platform. Data are mean ± SEM from 8 mice in each group. **c**, **e–g**, **i,** and **j** *P < 0.05 vs. WT mice. ^#^*P <* 0.05 vs. *APP/PS1* Tg mice (**i**). *P < 0.05 vs. day 1. ^*#*^*P <* 0.05 vs. WT mice

To assess the role of sEH on the impairment of AD-related behaviors in *APP/PS1* Tg mice, we generated the *APP/PS1* Tg/sEH^−∕−^ mice by an inbred of *sEH*^−∕−^ mice with *APP/PS* Tg mice (Fig. 3a). The analysis of nesting building activity showed that *APP/PS1* Tg/*sEH*^−∕−^ mice constructed higher quality of nest shape along with increased quantification of score compared with age-matched 6-month-old *APP/PS1* Tg mice (Fig. 3b and c). These data indicated that ablation of *sEH* improved the nesting building ability in *APP/PS1* Tg mice. In addition, spatial learning and memory were examined with Y-maze and MWM. In Y-maze test, loss of *sEH* expression increased the preference of first entry to new arm, the entry numbers ration, and distance in new arm in 6-month-old *APP/PS1* Tg mice (Fig. 3d–g). Next, the MWM test was performed to evaluate the ability of spatial learning and memory of mice. With the hidden platform test, *APP/PS1* Tg mice learned the location of the hidden platform since day 3, but *APP/PS1* Tg/*sEH*^−∕−^ mice learned the hidden platform location since day 2 (Fig. 3h). However, there were no statistically significant differences between *APP/PS1* Tg mice and *APP/PS1* Tg/*sEH*^−∕−^ mice in escape latency during the 4 days of training. Moreover, *APP/PS1* Tg/*sEH*^−∕−^ mice showed an increased number of times crossing the hidden platform and retention times in the target quadrant at day 5 after training (Fig. 3i and j). The genotypes did not differ in swimming to the visible platform test (Fig. 3k). These findings suggest that the sEH might be a critical regulator in AD-related cognitive performance and spatial learning and memory ability.

### Loss of sEH not only diminishes Aβ deposition, but also decreases apoE expression in brain lesions

**Fig. 4:**
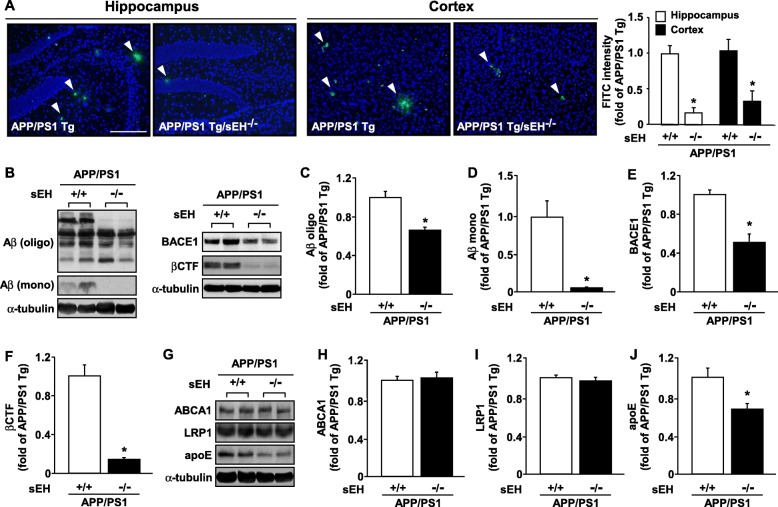
**a–j** Loss of function of *sEH* decreases Aβ deposition in *APP/PS1* Tg mice. **a** Brain tissue sections from 6-month-old *APP/PS1* Tg and *APP/PS1* Tg/*sEH*^−∕−^ mice were immunostained with anti-Aβ and FITC-conjugated secondary antibody, and then the FITC intensity was assessed. Scale bar = 100 μm. **b**–**j** Western blot analysis of protein levels of oligomer (oligo) or monomer (mono) Aβ, BACE1, βCTF, ABCA1, LRP-1, apoE, and α-tubulin. Data are mean ± SEM from 8 mice in each group. *P < 0.05 vs. *APP/PS1* Tg mice

Next, we further examined the level of Aβ plaque formation with or without sEH under *APP/PS1* Tg background. Immunohistochemistry analysis indicated that the Aβ accumulation was dramatically decreased on hippocampus and cortex of *APP/PS1* Tg/ *sEH*^−∕−^ mice (Fig. 4a). Similar results of decreased Aβ levels were obtained by western blotting analysis in the brain lysates of *APP/PS1* Tg mice and *APP/PS1* Tg/*sEH*^−∕−^ mice. Western blot results of Aβ in both oligomers and monomers, BACE1, and βCTF, within brain lysates from *APP/PS1* Tg mice and *APP/PS1* Tg/*sEH*^−∕−^ mice supported the decreased Aβ plaque formation in *APP/PS1* Tg/*sEH*^−∕−^ mice (Fig. 4b–f). Further, the levels of apoE, but not ABCA1 and LRP-1, were decreased in *APP/PS1* Tg/*sEH*^−∕−^ mice (Fig. 4g–j).

### Genetic ablation of *sEH* in an *APP/PS1* Tg background aggravates astrogliosis and increases the production of astrocyte-derived cytokines

**Fig. 5:**
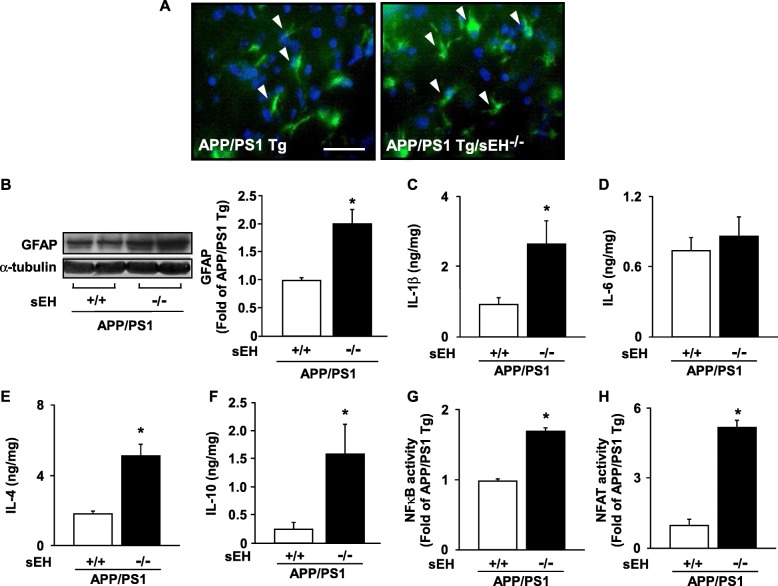
Loss of function of *sEH* increases astrogliosis and the production of cytokines in *APP/PS1* Tg mice. **a** Brain tissue sections from 6-month-old *APP/PS1* Tg and *APP/PS1* Tg/*sEH*^−∕−^ mice were immunostained with anti-GFAP (for astrocyte) and FITC-conjugated secondary antibody. Scale bar = 50 μm. **b** Western blot analysis of protein levels of GFAP and α-tubulin in the brain. **c**–**f** ELISA of the production of IL-1β, IL-6, IL-4, and IL-10 in brain specimens. **g, h** The DNA-binding activity of NF-κB and NFAT in brain specimens. Data are mean ± SEM from 8 mice in each group. *P < 0.05 vs. *APP/PS1* Tg mice

**Fig. 6:**
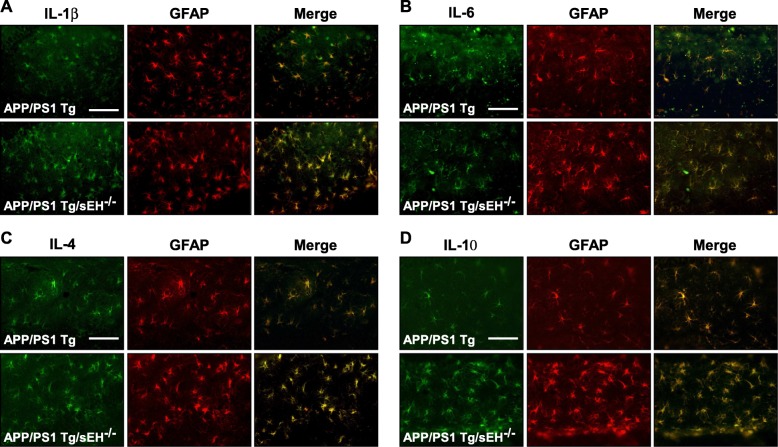
Astrocyte-derived cytokine production is increased in *APP/PS1* Tg/*sEH*^−∕−^ mice. **a**–**d** Immunostaining of hippocampus specimens from 6-month-old *APP/PS1* Tg and *APP/PS1* Tg/*sEH*^−∕−^ mice with anti-IL-1β, IL-6, IL-4, or IL-10 and anti-GFAP antibodies, then FITC- or Texas Red-conjugated secondary antibody. Scale bar = 50 μm. IL-1β-, IL-6-, IL-4-, and IL-10-positive astrocytes (green color) and GFAP-positive cells (red color) were co-localized, respectively

Subsequently, we observed the unexpected phenomena that the level of astrogliosis was more obvious in *APP/PS1* Tg/*sEH*^−∕−^ mice than that in *APP/PS1* Tg mice (Fig. 5a and b). However, deficiency of sEH markedly increased the production of pro-inflammatory cytokine IL-1β, and anti-inflammatory cytokines IL-4 and IL-10 in *APP/PS1* Tg mice (Fig. 5c–f). We next focused on the activities of transcription factor NF-κB and NFAT, which predominantly regulate the expression of the *IL-1β*, *IL-6*, *IL-4*, and *IL-10* genes [28–31]. We demonstrated that deletion of *sEH* increased the DNA-binding activity of NF-κB and NFAT in *APP/PS1* Tg mice (Fig. 5g and h). Next, immunohistochemistry analysis revealed that the increased levels of IL-1β, IL-4, and IL-10 were restricted in hippocampal astrocytes in *APP/PS1* Tg/sEH^−∕−^ mice (Fig. 6). Thus, loss of sEH may facilitate astrogliosis, but remarkably increase the production of anti-inflammatory cytokines IL-4 and IL-10 in *APP/PS1* Tg mice.

### Analysis of the gene ontology in *APP/PS1* Tg mice and *APP/PS1 Tg/sEH*^−∕−^ mice

In order to investigate the precise role of sEH in AD pathogenesis, we used LC-MS/MS and analyze gene ontology to address the possible pathway and mechanisms during AD progression. The quantitative proteomics analysis between the brain tissue samples of *APP/PS1* Tg/*sEH*^−∕−^ mice and *APP/PS1* Tg mice was performed following the LC-MS/MS method. Bioinformatics analyses of the deregulated proteins revealed significant pathways and processes known to be associated with AD progression. Upregulated proteins in *APP/PS1* Tg/*sEH*^−∕−^ mice are known factors that are involved in translation, oxidation, and cytoskeleton organization (Table 1). Moreover, downregulated proteins in *APP/PS1* Tg/*sEH*^−∕−^ mice indicated their key role in small GTPase-mediated signal transduction (Table 2). Thus, these findings provide the novel evidence that sEH plays a crucial role in regulating the biological process in AD pathogenesis.

**Table 1:** Gene ontological analysis: upregulation (APP/PS1 Tg/sEH^−∕−^ vs. APP/PS1 Tg)

Biological process	Count	Count *P* value
Generation of precursor metabolites and energy	14	3.77E−07
Translation	15	6.06E−07
Oxidation reduction	15	1.88E−03
Molecular functions		
Structural constituent of ribosome	10	7.76E−06
Nucleotide binding	38	4.21E−05
Cytoskeletal protein binding	14	7.72E−05
KEGG pathway		
Ribosome	9	2.83E−06
Oxidative phosphorylation	7	2.03E−03
AD	7	1.05E−02

The “Count” indicates the number of protein species whose expression has been altered in a defined biological process of the brain

**Table 2:** Gene ontological analysis: downregulation (APP/PS1 Tg/sEH^−∕−^ vs. APP/PS1 Tg)

Biological process	Count	*P* value
Positive regulation of protein complex disassembly	4	3.01E−05
Cellular macromolecular complex assembly	12	4.62E−05
Small GTPase-mediated signal transduction	13	4.81E−05
Molecular functions		
GTP binding	22	4.31E−09
Guanyl nucleotide binding	22	6.72E−09
Guanyl ribonucleotide binding	22	6.72E−09
KEGG pathway		
Ribosome	9	1.42E−04
FcγR-mediated phagocytosis	7	6.97E−03
AD	9	1.36E−02

## Discussion

In this study, we identified the detrimental effect of sEH in neuroinflammation and dysfunction of behavior outcomes in AD progression. The protein level of sEH was increased in 6-month-old *APP/PS1* Tg mice. Based on the immunohistochemistry results, we found that the increased levels of sEH in brain glial cells, especially in astrocytes. Besides, our findings that the loss of sEH retarded the progression of AD pathology by regulating Aβ plaque deposition, astrogliosis, and cytokines production, leading to the alleviation in the AD-impaired nesting building ability, spatial learning, and memory formation in *APP/PS1* Tg mice. Interestingly, we demonstrated that loss of sEH in *APP/PS1* Tg mice decreased Aβ plaque formation, but increased astrogliosis in the brain. Importantly, the production of anti-inflammatory cytokines and the activity of NF-κB and NFAT were also increased in *APP/PS1* Tg/*sEH*^−∕−^ mice, suggesting the crucial role of sEH in the regulation of neuroinflammation in AD pathology.

The quantitative proteomics analysis of brain tissue lysates from *APP/PS1* Tg mice and *APP/PS1* Tg/*sEH*^−∕−^ mice revealed that several significant pathways and cellular processes, including translational regulation, oxidative stress, and cytoskeleton reorganization, are possibly associated with the function of sEH as well as the pathogenesis of AD. Taken together, our results strongly suggest that sEH has an impact in regulating biological processes of the brain during the progression of AD pathology.

sEH is a bifunctional enzyme with EH and PT activities. One of the functions of sEH is to degrade cytochrome P450 products, such as arachidonic acid and epoxyeicosatrienoic acids (EETs) [1–4]. Recently, several lines of evidence suggest that the distribution of sEH plays an important role in the development of inflammatory diseases and brain diseases [1–4, 9–13]. Inhibition of the EH activity of sEH confers the protection from ischemia-induced brain injury [6, 7, 10–13]. Hung et al. reported that sEH in astrocytes is a critical regulator in inflammatory response brain injury in temporal lobe epilepsy [34], which implies that sEH is a crucial regulator in cerebrovascular and neuronal function upon the pathological insults. Additionally, Sarkar et al. addressed the detrimental effect of Aβ on degradation of EETs in the cerebellum, suggesting the possible involvement of sEH in AD pathology [16]. Moreover, Yao et al. showed that pharmacological inhibition of EH activity of sEH protects from tauopathy by regulating PI3K/AKT/GSK-3β pathway [35]. sEH has also been shown to be involved in age-related vascular cognitive decline [15]. Very recently, Ren et al. and Ma et al. reported that sEH plays a crucial role in the pathogenesis of Parkinson’s disease and neurodevelopmental disorders [36, 37]. These previous studies highlight the important role of sEH in the neurodegenerative diseases; therefore, the impact and detailed regulatory mechanism of sEH are worth elucidating. In this study, our findings further support this notion that deletion of *sEH* in *APP/PS1* Tg mice improved AD-related behavior outcomes and the progression of pathology.

APP can be rapidly metabolized by posttranslational proteolysis via an amyloidogenic or non-amyloidogenic pathway in neurons [38]. In the amyloidogenic pathway, APP is cleaved by BACE1 and results in the release of the N-terminal APP fragments and βCTF [38]. βCTF is then cleaved by γ-secretase to generate an amyloid precursor protein intracellular domain and a toxic Aβ peptide (37–49 amino acids), which triggers inflammatory responses of astrocytes and causes and neuron dysfunction [19, 23, 38]. On the other hand, the astrocyte-derived high-density lipoprotein (HDL)-like particles comprising cholesterol and apoE are known to play a central role in regulating neuron function and Aβ metabolism [39–41]. These secreted HDL-like particles can bind with insoluble β-amyloid, following uptake by glial cells through ABCA1 and LRP-1 to avoid the Aβ deposition [41]. Our data showed that the levels of Aβ peptides, BACE1, βCTF, and apoE were decreased in *APP/PS1* Tg/*sEH*^−∕−^ mice. However, the protein levels of ABCA1 and LRP-1 were not changed. These results suggest that the decreased levels of neurotoxic Aβ in the brain of *APP/PS1* Tg/*sEH*^−∕−^ mice could be attributed to a decrease in Aβ biosynthesis but not an increase in Aβ clearance. Nevertheless, the detailed mechanism underlying sEH-mediated regulation in regulating Aβ generation and its clearance needs further investigation.

Astrocyte-mediated inflammation has been shown to play a key role in the progression of AD pathology [25, 26, 42, 43]. Previous studies suggest that Aβ-elicited production of pro-inflammatory cytokines IL-1β and IL-6 is involved in the initiation and progression of AD [27, 44]. In contrast, anti-inflammatory cytokines IL-4 and IL-10 can limit inflammation by decreasing the production of pro-inflammatory cytokines during the development of AD [27]. In line with these findings, our findings demonstrated that the levels of anti-inflammatory cytokines, including IL-4 and IL-10, were increased in *APP/PS1* Tg/*sEH*^−∕−^ mice. We also found that deletion of *sEH* in *APP/PS1* Tg mice increased DNA-binding activity of NF-κB and NFAT, two key transcriptional factors for in the expression of IL-4 gene [28–31]. Thus, we suggest that improved AD-related behavior outcomes in *APP/PS1* Tg/*sEH*^−∕−^ mice were mediated by the increased anti-inflammatory responses. Nevertheless, further investigations are required for evaluating the exact mechanism underlying sEH-mediated inflammation and AD pathology.

Astrogliosis (referred to as reactive astrocytes) occurs prominently in the central nervous system in response to pathological insults [26, 45]. Although the biological significance of astrogliosis is not fully understood, reactive astrocytes are intimately associated with Aβ plaque and involved in the regulation of neural protection and repair, glial scarring, and neuroinflammation in the pathogenesis of AD [45]. In addition, the reactive astrocytes promote the degradation and clearance of Aβ in AD brain and consequently limit the formation of Aβ plaque [46]. Interestingly, we found greater astrogliosis in the brain of *APP/PS1* Tg/*sEH*^−∕−^ mice than that in *APP/PS1* Tg mice. In parallel, the levels of the anti-inflammatory cytokines were increased in the brain of *APP/PS1* Tg/*sEH*^−∕−^ mice. We thus suggest that sEH plays a critical role in regulating astrogliosis, which protects the neuronal cells from Aβ stimulation and delays the progression of AD.

To examine the possible molecular mechanism of sEH in AD pathogenesis, we used LC-MS/MS and gene ontology analysis to evaluate the involvement of sEH in cellular pathways during AD progression. Our results showed that the pathways of oxidative stress, cytoskeleton reorganization, small GTPase-mediated signal transduction, and translational regulation are regulated in the brain of *APP/PS1* Tg/*sEH*^−∕−^ mice as compared with that of *APP/PS1* Tg mice. Oxidative stress is an event that precedes the appearance of the pathologic hallmark of AD and forming a vicious cycle that promotes the initiation and progression of AD [47]. Besides tau protein, several histopathological cytoskeleton structures, including actin and the actin-binding protein, have recently been recognized in AD pathology [48]. Numerous studies showed that pathways governing actin cytoskeleton stability have deleterious effects on AD pathologies in human and mouse [48, 49]. The stability of actin cytoskeleton is important for regulating the normal structures and functions of dendritic spines [48–50]. For example, Rho GTPase, one of the small GTPase family proteins, acts as the key regulator in actin cytoskeleton remodeling and dendritic spine maintenance [50]. Overall, perturbed synaptic efficacy in dendritic spines finally causes the impairment of neuron functions in AD progression [47]. In addition, our data showed that genetic deletion of *sEH* deregulates the ribosome-related functions in *APP/PS1* Tg mice, which are agreement with Ding et al. who found that a posttranslational regulation-dependent impairment in ribosome function was detected in cortical areas of AD subjects [50].

Furthermore, several lines of evidence support the close relationship between oxidative stress and AD pathology [47, 51]. According to our LC-MS/MS results, the pathways of oxidative stress, cytoskeleton reorganization, small GTPase-mediated signal transduction, and translational regulation were modulated in *APP/PS1* Tg/*sEH*^−∕−^ mice compared with *APP/PS1* Tg mice. Our data also showed that *APP/PS1* Tg/*sEH*^−∕−^ mice exhibited improved AD-related behavior outcomes, Aβ production, and aggregation compared with *APP/PS1* Tg mice. Collectively, our findings suggested that sEH is critical in regulating the biological response of oxidative stress, cytoskeleton-dependent synaptic efficacy, and translational regulation during AD progression. However, extensive investigations are required for clarifying the participation and the regulatory mechanisms of sEH in these signaling pathways.

Nevertheless, our study contains several limitations because we only examined the effect of genetic deletion of sEH on the progression of AD pathology. We did not examine the levels of epoxide fatty acids in the brain of *APP/PS1* Tg mice and *APP/PS1* Tg/*sEH*^−∕−^ mice due to the technical limitation. In addition, from the point in search of drug targets, the roles of EH activity or PT activity of sEH in the progression of AD should be investigated by using pharmacological inhibitors targeting EH activity and PT activity of sEH in AD mice. To this end, further investigations describing the implications of sEH in AD are warranted.

## Conclusions

We provide several lines of novel evidence for the role of sEH in the pathogenesis of AD, which advances our knowledge for better understanding of the biological significance of sEH in AD. Our findings suggest a broader biological role of sEH and the underlying molecular mechanisms in the initiation and progression of neuroinflammatory and neurodegenerative diseases. Overall, sEH might be a potential pharmacological target for treating AD and related neurological diseases.

## Data Availability

The datasets used in the current study are available from the corresponding author on reasonable request.

## Abbreviations

ABCA1: ATP-binding cassette transporter A1
AD: Alzheimer’s disease
apoE: Apolipoprotein E
APP: Amyloid precursor protein
Aβ: Amyloid-β
BACE1: β-site APP cleavage enzyme 1
EH: hydrolase
GFAP: Glial fibrillary acidic protein
IBA-1: Ionized calcium-binding adapter molecule 1
IL: Interleukin
LC-MS/MS: Liquid chromatography-tandem mass spectrometry
LRP-1: LDLR-related protein 1
NFAT: Nuclear factor of activated T cells
NF-κB: Nuclear factor-κB
PS1: Presenilin 1
PT: Phosphatase
sEH: Soluble epoxide hydrolase
vWF: von Willebrand factor
βCTF: β-APP C-terminal fragment
